# Postoperative Rehabilitation after Anterior Cruciate Ligament Reconstruction through Telerehabilitation with Artificial Intelligence Brace during COVID-19 Pandemic

**DOI:** 10.3390/jcm12144865

**Published:** 2023-07-24

**Authors:** Wei-Jen Liao, Kun-Tsan Lee, Liang-Yu Chiang, Che-Han Liang, Chao-Ping Chen

**Affiliations:** 1 Department of Orthopaedics, Taichung Veterans General Hospital, Taichung 40705, Taiwan; cavaliarjames@gmail.com (W.-J.L.);; 2 Department of Post-Baccalaureate Medicine, National Chung-Hsing University, Taichung 402202, Taiwan; 3 Department of Orthopaedic Surgery, Taichung Armed Forces General Hospital, Taichung 41152, Taiwan; 4 School of Medicine, National Defense Medical Center, Taipei 11490, Taiwan; 5 Department of Orthopaedics, Tungs’ Taichung MetroHarbor Hospital, Taichung 43503, Taiwan; 6 Department of Acupressure Technology, Jen-Teh Junior College of Medicine, Nursing and Management, Miaoli 35664, Taiwan; 7 Department of Health Services Administration, China Medical University, Taichung 406040, Taiwan

**Keywords:** anterior cruciate ligament reconstruction, telerehabilitation, knee brace, COVID-19

## Abstract

Background: Due to the rapid spread of the coronavirus disease-19 (COVID-19), most of the patients expressed a reluctance to undergo postoperative rehabilitation at a rehabilitation clinic. Therefore, in this scenario it was necessary to reshape the crucial role of postoperative rehabilitation of these patients. We conducted a telerehabilitation program based on an artificial intelligence brace (AI brace) which can monitor the progress of rehabilitation through an app and an internet server. Our hypothesis was that home-based telerehabilitation might provide clinical outcomes comparable to face-to-face, hospital-based rehabilitation programs in terms of effectiveness. Methods: A retrospective cohort study enrolled patients who received anterior cruciate ligament reconstruction (ACLR) between January and September 2020. Patients were divided into two groups: the tele-AI group received telerehabilitation with an AI brace while the FTF group had face-to-face, hospital-based rehabilitation. Clinical knee functional scores and Tegner Activity Scale (TAS) were assessed and analyzed until 12 months after the operation. Results: The tele-AI group had higher IKDC scores at 3 months (*p* = 0.0443) and 6 months (*p* = 0.0052) after surgery and higher KOOS scores at 1 month (*p* = 0.0365) and 6 months (*p* = 0.0375) after surgery. However, no significant difference between the two groups was detected at the end of the follow-up. The tele-AI group had higher TAS than FTF group after 1 year. Conclusions: Telerehabilitation after ACLR seems to provide a superior short-term outcome compared to hospital-based rehabilitation during the COVID-19 pandemic.

## 1. Introduction

Performing physical rehabilitation after anterior cruciate ligament reconstruction (ACLR) is crucial [1,2]. Out of various rehabilitation programs, postoperative bracing, which is designed to limit the range of motion of the operated knee and protect it against excessive varus and valgus stress, is regarded as less necessary relative to other modalities such as early weight-bearing, range-of-motion, strengthening, and functional exercises [1]. However, with the rapid spread of the 2019 novel coronavirus (COVID-19) [3], most patients have expressed a reluctance to undergo postoperative rehabilitation at a rehabilitation center. Therefore, the crucial role of postoperative rehabilitation must be re-evaluated.

Research has indicated that home-based telerehabilitation is just as effective as standard rehabilitation after a knee or hip replacement [4,5,6,7,8,9,10]. Our hospital has access to telerehabilitation services supported by an artificial intelligence (AI) brace, a portable sensor, and a built-in rehabilitation guidance system. This equipment can be used to implement home-based telerehabilitation, thereby eliminating the risk associated with visiting a hospital during the COVID-19 pandemic. Under the usage of telerehabilitation with AI brace, the rehabilitation progress of patients could be tracked by medical team members. Patients would be informed of the goal of rehabilitation by education sheets or the smart phone app. Rehabilitation progress can be recorded then uploaded to the cloud database. Once the performance is behind schedule, the system could alert the medical team and doctors can contact the patient directly by either phone or video consultation to solve the difficulties that patients could encounter. The popular application of video consultation makes telerehabilitation easier during the pandemic in our society. On the contrary, patients under telerehabilitation or face-to face program are able to contact our team if any problem occurs and obtain medical assistance immediately.

The possibility of telerehabilitation is attributed to the progress of telecommunication technology and delivering rehabilitation services remotely. The concept of telerehabilitation was first described in 1998 by Burns et al. [11]. The definition of telerehabilitation is the use of telecommunications technology to provide rehabilitation and long-term support to people with disabilities. This feature was initially used in cardiac and neurological rehabilitation. It allows patients to receive therapy and support from healthcare professionals without being physically present at a clinic or hospital. This approach has gained popularity due to its potential to increase accessibility, convenience, and cost-effectiveness. Nowadays, telerehabilitation has extended to many fields and different applications. Reviews of the result of telerehabilitation have generally been positive. Telerehabilitation eliminates geographical barriers, making it easier for individuals in remote areas or with limited mobility to access rehabilitation services. Telerehabilitation can potentially reduce healthcare costs by eliminating travel expenses and reducing the need for in-person appointments in the musculoskeletal field [12]. However, it is important to note that telerehabilitation may not be suitable for all individuals or conditions. Some cases may still require in-person assessment and hands-on therapy. The effectiveness of telerehabilitation can vary depending on the type of condition being treated and the specific therapy being provided.

To date, no study has compared the outcomes of AI-brace-assisted telerehabilitation programs and face-to-face, hospital-based rehabilitation programs conducted after ACLR. Our study aimed to compare the clinical outcomes of these two types of programs. Our hypothesis was that home-based telerehabilitation involving the use of an AI brace produces clinical outcomes comparable to that of face-to-face, hospital-based rehabilitation programs in terms of effectiveness.

## 2. Materials and Methods

### 2.1. Patient’s Population

The present retrospective cohort study was conducted at a single medical center in Taichung, Taiwan. We followed consecutive patients aged between growth plate maturation and epiphyseal fusion to 55 years old who underwent ACLR in our medical center by two experienced surgeons between January and September 2020. Patients enrolled in our study were not specified to a particular sports field. The other inclusion criteria were a preinjury Tegner Activity Scale (TAS) level of at least level 5, the use of a protective knee brace after ACLR, and the completion of scheduled outpatient department (OPD) follow-up visits (at least 12 months) postoperatively. The present study excluded patients who underwent revision surgeries, had multiple ligament injuries, or had concomitant comorbidities during the study period (e.g., osteochondral lesion (Outerbridge grade 3 or 4) and severe meniscal damage) and patients did not undergo regular follow-up for at least 12 months after their operation. All patients underwent arthroscopic ACLR, involving the use of autologous quadruple hamstring tendons. All surgical procedures were performed by a single experienced orthopedic surgeon, and their clinical outcomes were evaluated postoperatively at 1, 3, 6, and 12 months.

In total, 30 patients were enrolled; 15 patients underwent telerehabilitation assisted by an AI brace (Tele-AI group), and the other 15 underwent face-to-face, hospital-based rehabilitation (FTF group). The participants who underwent telerehabilitation used an AI brace with a built-in rehabilitation guidance system. The participants who underwent hospital-based rehabilitation (FTF group) did so at our institution’s rehabilitation center twice a week (at least once a week) for 3 months after their operation and once a week after 3 months until 6 months post-operation. The advantages and disadvantages of telerehabilitation were explained to the participants. Some participants preferred telerehabilitation because of the decreased number of required hospital visits during the COVID-19 pandemic and the fact that daily rehabilitation could be monitored directly by us through a web portal during the postoperative period. Patients in both the FTF group and the tele-AI group were asked to visit the outpatient department every month in the first three months. We followed up with a radiograph and collected postoperative clinical data after 6, 9, and 12 months. The participants were also provided information pertaining to home-based rehabilitation. Informed consent was obtained from all participants. The present study was performed in accordance with the Declaration of Helsinki. Approval was granted by the Institutional Review Board of Taichung Veterans General Hospital (July 29, 2021/CE21300B). The identification information of all participants was omitted prior to the analysis being conducted.

### 2.2. Rehabilitation Protocol

For all thirty patients in our study, we followed the post ACLR rehabilitation protocol set up by our hospital (Table 1)—partial weight bearing with a crutch in the first two weeks with general weight-bearing from the third week. Limited range of motion with knee brace and start with 0 to 45 degrees in the first two weeks. 0 to 60 degrees in the third week and 0 to 90 degrees in the fourth week under the knee brace. After 1 month, there is no restricted range of motion, and it is suggested that the knee brace is worn until three months. Jump rope begins 3 months postoperative and jogging on the uneven road starts 6 months postoperative. Return to sport is allowed at least 1 year after surgical intervention.

### 2.3. Home-Based Telerehabilitation

**Figure 1:**
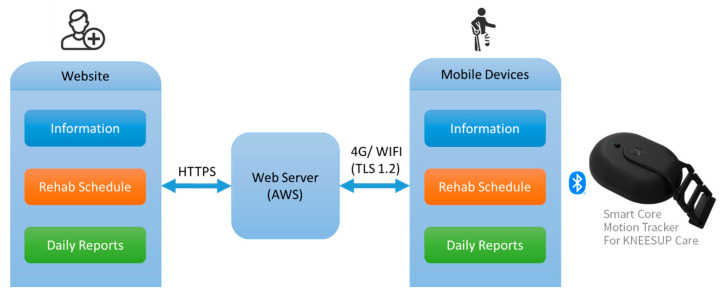
Patients conducted the daily rehabilitation at home by a mobile application and connected to the smart core motion tracker via Bluetooth. Orthopedic doctors or physical therapists could monitor the condition of each patient and provided suggestions.

The participants in the Tele-AI group underwent home-based rehabilitation through the rehabilitation system (KNEESUP Compact, Conzian Ltd., Taipei, Taiwan), which comprised a portable motion tracker, a mobile application (KNEESUP care, Conzian Ltd., Taipei, Taiwan), and a web portal (Figure 1). The app can be download from both Google Play and Apple store by a smart phone. Both the rehabilitation system and the mobile app were verified by verified by Industrial Technology Research Institute, Taiwan.

The participants used a mobile device that could be connected to a portable motion tracker through Bluetooth (Bluetooth 4.2 with the 10 Hz sampling frequency) to perform and track their daily rehabilitation. The smart core motion tracker (Conzian Ltd., Taipei, Taiwan) for AI brace was built with accelerometers, angle sensors, and gyroscopes. Thus, the smart core can track motion in three-dimensional space. The product is certified by Industrial Technology Research Institute, Taiwan.

A home-based rehabilitation schedule was designed by orthopedic doctors or physical therapists and set up on the web portal. Patients would download the rehabilitation program on the smart phone by the KNEESUP Care App (Conzian Ltd., Taipei, Taiwan). The app would supply the demonstration video to prevent inaccurate rehabilitation exercise and track the real-time motion by the sensor on the knee brace. Daily rehabilitation achievements were recorded and stored on a cloud-based database, allowing for orthopedic surgeons to follow up the status of each participant by accessing a web portal. Through this rehabilitation system, the conditions of the participants could be monitored, and their rehabilitation progress tracked. When a problem was detected, the affected patient was promptly provided medical support and the rehabilitation schedule can be modified individually.

### 2.4. Face-to Face Hospital-Based Rehabilitation

The participants in the FTF group underwent postoperative rehabilitation by visiting our hospital’s rehabilitation center twice a week (at least once a week) for 3 months after operation and once a week after 3 months until 6 months. The rehabilitation protocol is the same as the telerehabilitation group (Table 1). The frequency of visiting rehabilitation center was recorded. The goal of their rehabilitation program was to restore their level of activity to TAS level 5 (including heavy labor work, cycling, and jogging on uneven ground at least twice weekly) 6 months after surgery.

### 2.5. Clinical Outcome Evaluation

After the qualified patients were selected as participants, their knee function was clinically assessed and scored during patient visits. For all participants, data were collected before surgery (baseline data) and during OPD follow-up visits at 1, 3, 6, and 12 months after surgery. The collected data comprised International Knee Documentation Committee (IKDC) Subjective Knee Evaluation Form scores and knee injury and osteoarthritis outcome (KOOS) scores (comprising symptoms, pain, function in daily living, function in sports and recreational activities, and quality of life) [13]. Additionally, the TAS level of each participant (i.e., the level that most accurately described their current level of activity) was assessed at 12 months after the operation.

### 2.6. Statistics

Statistical analysis was performed to analyze the aforementioned variables of the 30 participants who completed at least 12 months of postoperative OPD follow-up. All analyses were performed using SPSS version 22.0 (IBM, NY, USA), and the results are represented as means and standard deviations. A *p*-value of <0.05 was regarded as statistically significant. Categorical variables were tested using the Fisher’s exact test and continuous variables were tested using the Mann–Whitney U test for intergroup comparisons. The Wilcoxon signed-rank test was performed for intragroup comparisons; specifically, the mean gross grading scores of all participants at 1, 3, 6, and 12 months after surgery were compared with their preoperative scores.

## 3. Results

The average age at surgery was 26.27 ± 8.59 years (range: 17–43 years) for the Tele-AI group and 28.60 ± 9.30 years (range: 17–50 years) for the FTF group. Of the 30 patients, the majority had a sports-related anterior cruciate ligament injury (86.7%). The preoperative participant demographics are presented in Table 2. All patients at least had follow-up for 1 year after the surgery.

In the FTF group, the frequency of visiting rehabilitation center after ACLR is 19.6 ± 2.2 (range: 16–23) times in the first twelve weeks and 9.7 ± 1.5 (range 8–12) times between months 4 and 6. Every patient in FTF group visits the rehabilitation center at least once a week and 6 (40%) patients visit the rehabilitation center below 18 times (75%) in the first twelve weeks. In addition, no patient gets infected with COVID-19 during the follow-up periods, and no outbreaks of cluster infection episodes happened in the rehabilitation center.

**Figure 2:**
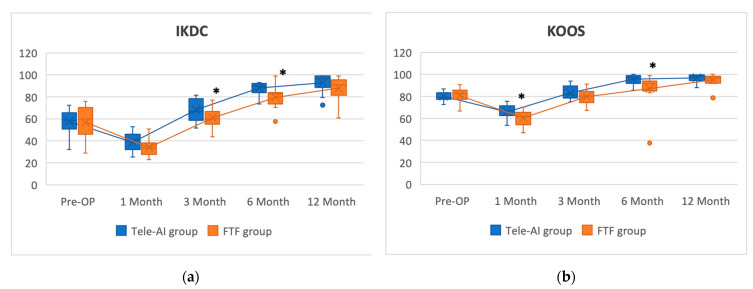
The intragroup and intergroup comparison of IKDC (**a**) and KOOS (**b**) from baseline to postoperative 12 months. *: Indicate comparable intergroup scores with significant difference (*p* < 0.05).

The patient-reported outcomes (i.e., IKDC and KOOS scores) are presented in Table 3 and Figure 2. No significant difference in baseline IKDC and KOOS scores was detected between the Tele-AI and FTF groups (IKDC, Tele-AI vs. FTF, 57.63 ± 10.95 vs. 56.78 ± 14.78, *p* = 0.983; KOOS, Tele-AI vs. FTF, 80 ± 3.91 vs. 80.71 ± 7.04, *p* = 0.405).

At 1 month after surgery, both groups had lower IKDC and KOOS scores than they did at baseline. Relative to the FTF group, the Tele-AI group had higher IKDC scores at 3 months (*p* = 0.0443) and 6 months (*p* = 0.0052) after surgery and higher KOOS scores at 1 month (*p* = 0.0365) and 6 months (*p* = 0.0375) after surgery. No significant difference between the two groups was detected at 12 months after surgery (*p* = 0.1568 and 0.2306 for Tele-AI and FTF groups, respectively).

**Figure 3:**
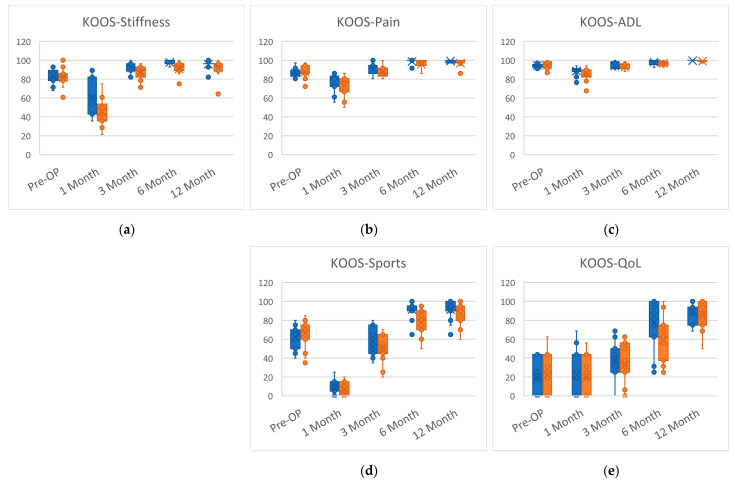
The intragroup and intergroup comparison of KOOS subscales from baseline to 12 months post-operation. (**a**) KOOS-Stiffness. (**b**) KOOS-Pain. (**c**) KOOS-ADL. (**d**) KOOS-Sports. (**e**) KOOS-QoL KOOS-Symptoms: Subscale of knee injury and osteoarthritis outcome score. Symptoms. KOOS-Pain: Subscale of knee injury and osteoarthritis outcome score. Pain. KOOS-ADL: Subscale of knee injury and osteoarthritis outcome score. Function in daily living. KOOS-Sport: Subscale of knee injury and osteoarthritis outcome score. Function in sports and recreational activities. KOOS-QoL: Subscale of knee injury and osteoarthritis outcome score. Quality of life.

The KOOS subscale outcomes are presented in Table 4 and Figure 3. For the KOOS subscales (KOOS-Symptoms, KOOS-Pain, KOOS-ADL, KOOS-Sport/Rec, and KOOS-QoL), no significant intergroup difference between the two groups was detected at baseline. Relative to their baseline scores, the two groups’ IKDC and KOOS scores were lower at 1 month after surgery but approximated the baseline levels at 3 months after surgery. KOOS-Symptoms, KOOS-Pain, KOOS-Sport/Rec, and KOOS-QoL were significant at 6 months post-ACLR. With the exception of KOOS-ADL, there was no significant difference between the tele-AI group and FTF group. However, no significant difference was detected at 12 months after surgery.

At 12 months after surgery, the Tele-AI group exhibited more favorable TAS results relative to the FTF group. Fourteen participants in the Tele-AI group (93.3%) and 11 participants in the FTF group (73.3%) reached TAS level of 5. In addition, five participants (33.3%) in the FTF group did not adhere to the rehabilitation program conducted at the rehabilitation center (i.e., did not visit the center regularly), and these five patients also had a relatively low TAS level.

## 4. Discussion

The purpose of this study was to compare the outcomes of an AI-brace-assisted telerehabilitation program to those of a face-to-face, hospital-based rehabilitation program; these programs were designed for patients who had undergone ACLR and were conducted during the COVID-19 pandemic. Relative to baseline, the Tele-AI group exhibited more favorable IKDC results at 3 and 6 months after surgery and more favorable KOOS results at 1 and 6 months after surgery. All participants were assumed to have complied with their assigned postoperative rehabilitation program (telerehabilitation or face-to face hospital-based rehabilitation). The compliance of the Tele-AI group was monitored directly through the web portal, and they were observed to have maintained a high level of compliance throughout the postoperative period. By contrast, more than one-third of the participants (6 of 15 patients, 40%) in the FTF group failed to regularly attend scheduled postoperative rehabilitation sessions at the rehabilitation center for fear of being infected with COVID-19.

At 6 months post-operation, our results showed significant IKDC and KOOS scores in the tele-AI group, which may contribute to the compliance of rehabilitation. To understand why the outcomes are inferior in the FTF group, three patients in FTF had especially poor short-term outcomes and they were also far behind our rehabilitation protocol due to their fear of the pandemic. According to Rodríguez-Merchán et al., a successful recovery from ACLR surgery is not only dependent on surgical technique and surgeon experience but also on postoperative rehabilitation [14]. In the present study, the unpredictable outcomes resulting from inconsistent adherence to the hospital-based rehabilitation program during the COVID-19 pandemic could have explained the less favorable results during the short-term postoperative period. However, the nonsignificant difference between the results at baseline and at 12 months after surgery may have been related to the ebbing of COVID-19 infections in Taiwan.

Whether a knee brace is required after ACLR is a debatable topic [1,14,15,16,17]. Rodríguez-Merchán et al. mentioned in his reviewed article that postoperative bracing after ACLR does not relieve pain and induce function and stability. Although most of the high-level evidence revealed post operative knee brace is not necessary, we can still use it as a tracker device for telerehabilitation. The AI brace used by the Tele-AI group not only provided the protective effects of an ordinary knee brace but also enabled us to monitor the patient compliance during the COVID-19 pandemic, which contributed to the predictable outcomes of the group.

Advancements in telecommunications technology have facilitated the implementation of internet-based telerehabilitation [18]. The definition of telerehabilitation was defined to increase the intensity and provide the continuity of rehabilitation after patient discharged. The clinical efficacy of telerehabilitation for orthopedic surgery (e.g., total knee arthroplasty, total hip arthroplasty, shoulder joint arthroplasty, and degenerative lumbar spine surgery) has been demonstrated to be superior or comparable to that of standard rehabilitation programs [7,8,10,19,20,21,22,23]. In addition, telerehabilitation after total knee arthroplasty or ACLR surgery has also been verified to be a cost-effective model [24,25]. Telerehabilitation provides a non-contact visit choice during the pandemic. Higgins et al. also demonstrated, in his randomized control trial, a decrease of in-person visits in the home app group compared to conventional rehabilitation groups [25]. The healthcare cost was also reduced in the home app group. Telerehabilitation provided a solution during the lockdown by allowing patients to maintain a safe distance and reduce unnecessary visits to the hospital, which was important in this tough period.

Bauwens et al. showed short-term results with 32 patients after ACLR surgery during the COVID-19 pandemic with self-rehabilitation guided by the app [26]. This limited the adverse effects and similar outcomes with standard rehabilitation in 6 months post-operation. However, our tele-AI group had better results after 6 months follow-up, but no significant findings remained at the final follow-up (post-ACLR 12 months). Bouguennec et al. reported that ACLR with self-guided rehabilitation during the pandemic increase the increasese of cyclops syndrome, which is related to the poor recovery after the surgery [27]. In our study, cyclops syndrome did not occur in the FTF group or in the tele-AI group during the 12 months follow-up period. We assumed that this may have been influenced by the small number of cases, and further study and evaluation should be arranged. There is no revision surgery required in both tele-AI group and FTF group during the follow-up.

A study by N.J. Collins et al. revealed that the KOOS-ADL subscale had better content validity for old patients with osteoarthritis undergoing total knee arthroplasty, while KOOS-Sport/Rec and KOOS-QoL had higher content validity for younger patients with ACL injury [28]. This could explain the non-significant results shown by using the KOOS-ADL subscale 6 months post-operation.

To the best of our knowledge, the present study is the first to evaluate the clinical outcomes of telerehabilitation after ACLR surgery during the COVID-19 pandemic. The Tele-AI group (telerehabilitation) attained more favorable results than the FTF group (standard rehabilitation) did during the COVID-19 pandemic. Despite that, the most perfectly designed telerehabilitation could only have a similar but not superior outcome compared to hospital-based rehabilitation. In our situation, the pandemic and the fear of getting infected with COVID-19 decreased the frequency of people visiting rehabilitation centers. Poor compliance generated unpredictable results.

However, the present study has several limitations. First, it adopted a retrospective cohort design, which is inherently susceptible to information bias. Second, the small sample of the present study did not allow for robust subgroup analyses to be performed, and the postoperative follow-up period was limited to 12 months after the index surgery. Third, the generalizability of the present study is low because it was conducted at a single institute. Finally, the high cost of the AI brace used in the present study may have affected the decision-making of patients and led to selection bias.

## 5. Conclusions

Our findings revealed that during the COVID-19 pandemic, AI-brace-assisted telerehabilitation produced results that were comparable to those of a standard rehabilitation program. Thus, the proposed telerehabilitation program can benefit patients who cannot regularly visit a rehabilitation center. Nevertheless, further studies are necessary to elucidate the benefits of AI-brace-assisted telerehabilitation followed by a regular postoperative rehabilitation program in a pandemic-free environment.

## Figures and Tables

**Table 1:** Rehabilitation protocol.

Post Operative Period	Rehabilitation Task
1–2 weeks	Range of motion 0–45 degrees Partial weight bearing with crutch Quadriceps muscle isometric contraction Straight leg raise Ankle pumping
3 weeks	Range of motion 0–60 degrees Full weight bearing as tolerated Gait and balance training Passive stretch for knee extension quadriceps Heel slide on wall
4 weeks	Range of motion 0–90 degrees Passive stretch for knee extension in prone position Patella mobilization Semi-squat and heel-up
5–8 weeks	Range of motion not restricted Wall squat Stairs up and stairs down
8–12 weeks	Range of motion not restricted Plunk and bridging exercise Single leg squat Single leg stands with eyes closed Advanced stairs up and stairs down
12–16 weeks	Jump rope Jogging straight on the even road
16–24 weeks	Advanced stairs up and stairs down Advanced single leg squat Single leg crossing cone reach Jump and single leg land Single leg hops in place
After 24 weeks	Jogging on the uneven road or jogging with turns Acceleration or deceleration running Sports specific activity

**Table 2:** Characteristics of preoperative patients.

	Tele-AI Group	FTF Group	*p* Value
No.	15	15	
Age at surgery (years)			
Mean ± SD	26.27 ± 8.59	28.60 ± 9.30	0.482
Median	24	26	
Range	17–43	17–50	
No. of male/female patients	11/4	10/5	0.787
No. of right/left injuries	8/7	7/8	0.608
No. of sports/traumatic injuries	13/2	13/2	1.000

No.: number, SD: standard deviation.

**Table 3:** Intergroup comparison of IKDC and KOOS from baseline to postoperative 12 months.

	Tele-AI Group	FTF Group	
	Mean ± SD	Mean ± SD	*p* value
IKDC			
Pre-OP	57.63 ± 10.95	56.78 ± 14.78	0.2738
Post-OP 1 m	38.62 ± 8.73	34.01 ± 8.16	0.1467
Post-OP 3 m	68.21 ± 9.66	60.77 ± 9.67	0.0443 *
Post-OP 6 m	87.84 ± 5.55	79.49 ± 9.11	0.0052 *
Post-OP 12 m	92.67 ± 8.27	87.83 ± 9.85	0.1568
KOOS			
Pre-OP	80 ± 3.91	80.71 ± 7.04	0.734
Post-OP 1 m	65.85 ± 6.57	60.56 ± 6.61	0.0365 *
Post-OP 3 m	83.18 ± 6.13	79.77 ± 6.02	0.1353
Post-OP 6 m	95.59 ± 4.19	87.13 ± 14.40	0.0375 *
Post-OP 12 m	96.9 ± 32.8	94.9 ± 5.38	0.2306

* Represents there is significant difference between [Tele-AI group] and [FTF group]. SD: standard deviation. IKDC: International knee documentation committee. KOOS: Knee Injury and osteoarthritis outcome score. AI: Artificial intelligence. Pre-OP: Preoperative baseline. Post-OP 1 m: Postoperative 1 month. Post-OP 3 m: Postoperative 3 months. Post-OP 6 m: Postoperative 6 months. Post-OP 12 m: Postoperative 12 months.

**Table 4:** Intergroup comparison of KOOS subscales from baseline to 12 month post-operation.

	Tele-AI Group	FTF Group		Tele-AI Group	FTF Group		Tele-AI Group	FTF Group	
	Mean ± SD	Mean ± SD	*p* Value	Mean ± SD	Mean ± SD	*p* Value	Mean ± SD	Mean ± SD	*p* Value
	KOOS-Symptoms			KOOS-Pain			KOOS-ADL		
Pre-OP	83.33 ± 7.72	81.43 ± 10.39	0.571	86.67 ± 4.72	87.41 ± 6.71	0.478	94.61 ± 2.13	94.81 ± 4.41	0.413
Post-OP 1 m	60.24 ± 18.65	45.24 ± 14.01	0.024 *	77.59 ± 9.71	72.96 ± 11.47	0.134	88.63 ± 4.39	85.98 ± 6.92	0.221
Post-OP 3 m	91.91 ± 6.11	86.91 ± 8.17	0.093	90.37 ± 6.71	87.04 ± 5.52	0.14	93.92 ± 3.51	92.75 ± 3.21	0.239
Post-OP 6 m	97.62 ± 2.58	90.95 ± 6.87	0.001 *	98.70 ± 2.75	95.74 ± 4.05	0.020 *	97.94 ± 2.65	96.77 ± 2.37	0.093
Post-OP 12 m	95.8 ± 4.31	91.9 ± 8.69	0.146	99.26 ± 4.31	96.85 ± 4.68	0.096	99.61 ± 0.87	99.02 ± 1.98	0.334
	KOOS-Sport/Rec			KOOS-QoL					
Pre-OP	62.33 ± 11.63	66.33 ± 15.17	0.178	19.17 ± 18.37	22.50 ± 21.62	0.68			
Post-OP 1 m	10.00 ± 6.81	6.33 ± 7.19	0.183	22.08 ± 22.76	19.17 ± 20.66	0.777			
Post-OP 3 m	58.00 ± 14.12	50.33 ± 14.07	0.24	37.50 ± 19.48	32.50 ± 21.29	0.537			
Post-OP 6 m	91.33 ± 8.96	78.00 ± 13.99	0.004 *	77.08 ± 28.02	58.75 ± 23.36	0.039 *			
Post-OP 12 m	91.33 ± 10.43	87 ± 11.62	0.359	89.17 ± 10.42	86.67 ± 14.92	0.618			

* Represents there is significant difference between [Tele-AI group] and [FTF group]. AI: Artificial intelligence. SD: standard deviation. Pre-OP: Preoperative baseline, Post-OP 1 m: Postoperative 1 month, Post-OP 3 m: Postoperative 3 months, Post-OP 6 m: Postoperative 6 months, Post-OP 12 m: Postoperative 12 months. KOOS-Symptoms: Subscale of knee injury and osteoarthritis outcome score—Symptoms. KOOS-Pain: Subscale of knee injury and osteoarthritis outcome score—Pain. KOOS-ADL: Subscale of knee injury and osteoarthritis outcome score—Function in daily living. KOOS-Sport/Rec: Subscale of knee injury and osteoarthritis outcome score—Function in sports and recreational activities. KOOS-QoL: Subscale of knee injury and osteoarthritis outcome score—Quality of life.

## Data Availability

Due to privacy and proprietary natures, participants of this study did not agree for their data to be shared publicly, so supporting data and code are not available.
